# Aortic and cardiovascular remodelling after thoracic endovascular aortic repair for blunt traumatic aortic injury in younger patients: A narrative review of physiological and clinical outcomes

**DOI:** 10.1113/EP092615

**Published:** 2025-06-09

**Authors:** Marco David Bokobza De la Rosa, Matti Jubouri, Thurkga Moothathamby, Mohamed Refaie, Ali Murtada, Idhrees Mohammed, Ian M. Williams, Damian M. Bailey, Mohamad Bashir

**Affiliations:** ^1^ Lancaster Medical School University of Lancaster Lancaster UK; ^2^ Hull York Medical School University of York York UK; ^3^ Barts and the London School of Medicine and Dentistry Queen Mary University of London London UK; ^4^ Department of General Surgery Ysbyty Glan Clwyd, Rhyl Wales; ^5^ Institute of Cardiac and Aortic Disorders (ICAD) SRM Institutes for Medical Science (SIMS Hospital) Chennai India; ^6^ Department of Vascular Surgery University Hospital of Wales Cardiff UK; ^7^ Neurovascular Research Laboratory, Faculty of Life Sciences and Education University of South Wales Pontypridd UK

**Keywords:** aortic physiology, aortic remodelling, aortic surgery, BTAI, cardiothoracic, TEVAR

## Abstract

Blunt traumatic aortic injury (BTAI) is severe, often fatal in younger populations due to high‐energy deceleration mechanisms. Thoracic endovascular aortic repair (TEVAR) has revolutionised BTAI treatment, surpassing the previously standard open surgical repair in mortality and complication rates. Despite its success, concerns arise regarding TEVAR's long‐term effects, especially in younger BTAI patients. Key physiological changes following TEVAR include alterations in aortic size, shape, compliance and flow dynamics, leading to loss of the Windkessel effect and a consequent increased pulse wave velocity and decreased radial strain, which can contribute to the development of hypertension. These alterations also predispose patients to changes in cardiovascular flow (increased reverse systolic flow, reduced maximum velocity and altered helical flow), potentially increasing the risk of left ventricular dysfunction and coronary artery disease. Physiological changes also increase the likelihood of complications such as graft migration. Clinical outcomes of TEVAR for BTAI have generally been favourable, with significant reductions in mortality and cerebrovascular accident rates compared to open surgical repair. However, long‐term complications, including the need for re‐interventions, remain a concern, though studies suggest these are infrequent. The durability of TEVAR in younger patients, who may experience decades of device use, poses unique challenges, particularly due to the natural progression of aortic morphology over time. Therefore, adapting TEVAR to the physiological needs of younger BTAI patients is essential. Developing more compliant endografts and using shorter stents with improved materials can help minimise structural and haemodynamic changes and enhance cardiovascular outcomes, supporting the long‐term health of this vulnerable population.

## Abstract (highlights)

- **What is the topic of this review?**
  How thoracic endovascular aortic repair (TEVAR) influences aortic morphology, haemodynamics and cardiovascular outcomes in younger blunt traumatic aortic injury (BTAI) patients, and the associated long‐term complications and mitigation strategies.
- **What advances does it highlight?**
  TEVAR alters aortic compliance and flow dynamics, increasing risks of hypertension, cardiac remodelling, and complications like endograft migration and endoleaks. However, TEVAR offers low mortality and complication rates versus open surgical repair. Strategies such as reducing stented aortic length, improving endograft materials, and precise device sizing are critical for mitigating risks, enhancing outcomes and addressing the unique physiological demands of younger BTAI patients.

## 1 INTRODUCTION

Blunt traumatic aortic injury (BTAI) is a devastating vascular lesion with markedly high mortality rates, culminating in a pre‐hospitalisation death of 80%, making BTAI the second leading cause of death in the young, only second to head injuries (Scalea et al., 2019). These patients, commonly under 50 years of age, often present with non‐specific symptoms such as hypotension, altered mental state and evidence of external trauma; injuries involve a brisk deceleration mechanism, typically following motor vehicle accidents. The aortic isthmus is the most common site for BTAI, with damage to this area accounting for over 50% of the cases (Feczko et al., 1992). The aortic isthmus describes a transition zone between the mobile aortic arch and the anchored descending thoracic aorta; this change in range of movement along with its weaker tensile resistance and its location between two bony structures make the isthmus particularly susceptible to deceleration injuries. (Mouawad et al., 2020).

The approach for BTAI treatment has been revolutionised over the last decade, with thoracic endovascular aortic repair (TEVAR) becoming the gold‐standard strategy over the previously recommended open surgical repair (OSR). TEVAR was first approved in 2005 for treating thoracic aneurysm repair, but has since seen its potential usage amplified. The Society for Vascular Surgery 2011 guidelines first demonstrated that TEVAR was the preferred intervention in BTAI, obtaining better survival outcomes than OSR along with decreased complications, with patients experiencing less spinal cord ischaemia, kidney injury and infection (Lee et al., 2011; Makaroun et al., 2005). Even though TEVAR is now the treatment of choice for BTAI, several issues still arise with the utilisation of stents to manage these complex and dynamic injuries. TEVAR often struggles with arch conformation, often requiring the occlusion of the left subclavian artery (LSA) to guarantee a satisfactory seal in the proximal landing zone. LSA coverage can result in its own complications, increasing the risk of limb ischaemia, stroke and possible paraplegia stemming from the disruption of the collateral perfusion of the spine, as well as requiring LSA revascularisation in half of the cases (Gable et al., 2018).

The usage of TEVAR on a younger population than was initially designed for puts its adaptability and longevity to the test. It is important to note that the natural morphological changes that the aorta undergoes through ageing questions the adequacy of endografts in a young population. In addition, the endografts induce marked morphological and physiological changes in the cardiovascular system, which may accelerate vascular ageing, alter haemodynamics, and contribute to complications affecting both the stented segment and distal organs. Moreover, the younger age of BTAI patients means a longer follow‐up strategy is warranted, usually stretching over several decades along with repeated imaging radiation exposure, posing its own risks (Komutrattananont et al., 2019). Hence, the increasing popularity of TEVAR in managing BTAI has seen a rise in younger patients with thoracic stents, raising concerns for TEVAR's limitations in this population and its adverse effects on the circulatory system and target organs.

Ageing instigates alterations within aortic tissue compositions, changing structural properties and shape. The aorta experiences lengthening, wider luminal diameters, thicker inner layers, reduced elasticity and an increased collagen content as ageing occurs (Xu et al., 2017). These complicated morphological changes that occur in younger populations are not compensated for or replicable with any current stent on the market, querying the suitability of long‐term endovascular stents. Therefore, this narrative review aims to investigate the aortic and cardiovascular remodelling in BTAI patients treated with TEVAR, with specific emphasis on the physiological in vivo and in vitro evidence. An overview of postoperative clinical outcomes following TEVAR in BTAI is also presented.

## 2 PHYSIOLOGICAL EVIDENCE: AORTIC AND CARDIOVASCULAR REMODELLING

The increasing use of TEVAR for BTAI, particularly in younger populations, reflects the mechanisms of injury commonly associated with this condition. Boutin et al. (2022) observed 209 BTAI patient admissions over a 6 year period and reported that the average age of BTAI patients was 43.4 years, with motor vehicle‐related accidents accounting for 69% including car accidents (29% of total), pedestrian accidents (8% of total) and motorcycle accidents (30% of total), falls over 6 m accounted for 30% of cases, with other causes such as skiing or bicycle accidents accounting for the remaining 1%.

The young patient demographic raises important considerations about the long‐term physiological effects of TEVAR on the aorta and cardiovascular system. Unlike older populations, younger patients generally have more compliant aortas, fewer comorbidities and a lower overall cardiovascular risk profile (Bulpitt et al., 1999), making them healthier than the cohort for whom endografts were originally designed. TEVAR was initially designed to treat aneurysms in the descending aorta (Nation and Wang, 2015). A study by Gallo et al. (2023) compared the demographics of patients with chronic aneurysmal disease to those with traumatic aortic injury, finding that patients with aneurysmal disease were, on average, over 23 years older and exhibited nearly twice the likelihood of comorbid conditions. Specifically, rates of smoking (59% vs. 33%), hypertension (87% vs. 33%), diabetes (21% vs. 11%) and hypercholesterolaemia (78% vs. 44%) were significantly higher among aneurysmal patients than in those with traumatic injuries.

The increasing use of TEVAR for BTAI in younger patients, who generally exhibit fewer comorbidities, raises important questions about the long‐term effects of this intervention. How do the more compliant aortas and lower cardiovascular risk profiles of younger patients influence their response to the rigid endografts used in TEVAR? What unforeseen physiological effects might arise in this demographic, which differs significantly from the older populations for whom TEVAR was originally designed? Moreover, how might altered aortic remodelling and haemodynamic changes impact their overall cardiovascular health? Exploring these questions is crucial to understanding the implications of TEVAR in younger BTAI patients.

The application of TEVAR in this younger demographic warrants careful attention. Emerging research highlights the significant impact of TEVAR on aortic and cardiovascular anatomy and physiology, focusing on changes in aortic size, shape, compliance and flow dynamics, and their broader effects on the cardiovascular system.

### 2.1 Haemodynamic aortic remodelling

Aortic remodelling post‐TEVAR is a product of the mismatch between the native aorta and endograft. The aorta functions as a reservoir, storing approximately 50% of the left ventricular stroke volume during systole. During diastole, the elastic recoil of the aortic wall propels this stored blood forward into the peripheral circulation. This interaction between the blood ejected from the heart and the aortic compliance is known as the Windkessel effect, which plays a crucial role in maintaining continuous blood flow during the cardiac cycle (Sultan et al., 2021). With TEVAR the aorta loses compliance, and with it losing its ability to conform to the Windkessel effect (de Beaufort et al., 2017; Stonko et al., 2023). The reduction in the Windkessel effect means there is a reduction in the pulse wave propagation resulting in an undamped cardiac pulse which gives rise to greater pulse wave velocities (PWV) (Hori et al., 2020; Kadoglou et al., 2012; Takeda et al., 2014).

PWV is the speed at which pressure waves move through the blood vessels, it is directly linked with the elasticity of the aortic wall. Hence increased PWV is observed in individuals with less complaint and stiffer aortas where less pulse wave damping occurs (Ibrahim et al., 2010). An *ex vivo* porcine study by Nauta et al. (2016) observed how TEVAR halved radial strain in the stented areas, directly associating the increased PWV to TEVAR. This finding was further corroborated by Hori et al. (2021), who reported that patients with thoracic aortic aneurysms treated with TEVAR experienced an increase in PWV, regardless of the treatment site. The study by Nauta et al. (2016) also observed reduction in radial strain in the proximal segments to the endograft (*P* = 0.06) but not in the distal segments, indicating that TEVAR induced proximal radial stiffening, which has been linked to elevated wall stress and weakening of the aortic wall.

The elevated PWV observed post‐TEVAR has been tied to alterations in systolic blood pressure (SBP) and pulse pressure (PP) in young patients (Tzialis et al., 2012). Tzilalis et al. investigated PWV, reflected wave velocity and blood pressure alterations post‐TEVAR in patients with thoracic aortic transections. They hypothesised that the elevated PWV contributed to the rise in SBP observed in their study. This observation can be explained relating back to the Windkessel effect, where the loss of PWV propagation as a result of the stiffer endograft results in the higher PWV described. Tzialis et al. hypothesise that the increased PWV along with the stiffer segments of the aorta post‐TEVAR disrupt the normal waves that reflect back to reach the ascending aorta during diastole and which allow cardiac perfusion. The faster flow of blood is hypothesised to return earlier than normal, fusing with the end of systole, rather than during diastole, creating a later peak of arterial pressure and omitting diastole, hence increasing SBP and decreasing diastolic blood pressure (Hirata et al., 2006). These ideas are reflected in the literature. The study of Guala et al. (2023) investigated 26 BTAI patients (mean age 43.5 years) treated with TEVAR; they noticed new onset hypertension in 65% of the patients, and associated hypertension with a more proximal landing zone. Additionally Kamenskiy et al. (2020) looked at 20 patients treated with TEVAR for BTAI (mean age 34.9 years) and observed hypertension in 50% of their patients 5 years after the repair compared to 5% pre‐operatively. Gil‐Sala et al. (2021) also observed an increased PWV in the ascending aorta with increased aortic stiffness post TEVAR. These findings suggest that increased PWV following TEVAR may substantially elevate hypertension rates post‐procedure, particularly when proximal landing zones are used.

Tigkiropoulos et al. (2018) only found new onset hypertension in 34.8% of the patients post‐TEVAR, but linked new‐onset hypertension to younger age (a mean average of 15 years younger) and left subclavian artery coverage, with *P*‐values of 0.027 and 0.01, respectively. The increased susceptibility observed in younger patients may be explained by the enhanced aortic remodelling in this group, as described in Canaud et al. (2015). Canaud et al. investigated TEVAR outcomes in acute traumatic transections of the aorta. Their paper observed patients under the age of 30 at the time of intervention to experience significantly greater diameter net increases compared to their older counterparts.

The elevated PWV and BP described post‐TEVAR (Nauta et al., 2016; Tzialis et al., 2012) along with the associated aortic wall stiffening alters baroreceptor sensitivity and endothelial function. Baroreceptors, located in the aortic arch and carotid sinus, detect changes in arterial wall stretch to regulate blood pressure via the autonomic nervous system. When stiffness increases, as seen with TEVAR endografts, baroreceptor firing is blunted, leading to a compensatory increase in sympathetic outflow and subsequent hypertension, as reported in studies like Tzilalis et al. (2012) and Guala et al. (2023).

Along with an increased PWV, several studies observed an increased systolic reverse flow, along with a decreased maximum velocity (Aghilinejad et al., 2022; Garcia Reyes et al., 2019; Gil‐Sala et al., 2021; Girardin et al., 2024). These changes can be attributed to the junction between the endograft and native aorta; studies such as Sultan et al. (2022) and Cao et al. (2022) have described this point as a significant transition point in both shape and compliance. The abrupt change in vessel properties alters blood flow dynamics. The stiffer endograft segment can accept a more limited blood volume acting as a bottleneck; this slows down the blood flow as it enters the grafts, which may lead to localised flow obstruction and systolic flow reversal (Cosset et al., 2022). These changes, in conjunction with endograft disruption of the natural haemodynamic helical flow pattern, result in a reduction of maximum velocity (Morbiducci et al., 2009). The alterations in systolic reverse flow and maximum velocity are further influenced by structural changes in the aortic arch post‐TEVAR, which will be discussed in a following section.

Overall the endograft‐related haemodynamic changes stem from changes in wall compliance, size and shape. These changes result in a reduced pulse propagation, increasing PWV, and dampened baroreceptor sensitivity, as well as localised obstruction in the endograft proximal landing zone, reducing maximum wave velocity and increasing systolic flow reversal. These haemodynamic changes are directly related to an increased total vascular resistance, the resistance offered by both the systemic and pulmonary circulation. The alterations in blood flow also predispose patients who have undergone TEVAR for BTAI to increased systolic blood pressure through the loss of the Windkessel effect (Agrafiotis et al., 2023). Due to the younger age of patients with BTAI, the haemodynamic impact of TEVAR in this group is particularly significant, as the procedure's effects on vascular compliance and increased vascular resistance may accelerate cardiovascular ageing. The resulting haemodynamic stress, combined with the substantial flow alterations TEVAR induces, poses a heightened risk of long‐term complications such as hypertension and its related consequences, underscoring the need for careful monitoring and tailored post‐operative management in younger patients.

### 2.2 Morphological aortic remodelling

Aortic remodelling, including increases in both length and diameter, is a well‐documented phenomenon following TEVAR for BTAI.

Studies, including those by Ghazy et al. (2023) and Vallerio et al. (2019), have demonstrated the long‐term impact of TEVAR on the diameter of the ascending aorta, reporting average increases of 2.6 mm and 2.1 mm, respectively. Kamenskiy et al. (2020) further described consistent TEVAR impact, documenting a 2.4‐fold diameter expansion rate of the ascending aorta in BTAI patients treated with TEVAR compared to an age‐matched control group (Operated: 0.60 ± 0.80 mm/year; Control: 0.25 ± 0.44 mm/year). These results highlight the ascending aorta's heightened susceptibility to remodelling following TEVAR.

An increase in aortic diameter has been attributed to the effects of systolic reverse flow. A study by Weiss et al. (2023) investigated the association of reverse systolic flow in bicuspid aortic valves with aortic dilatation. They observed an independent relation between mid‐aorta ascending diameter and systolic reverse flow; however, they theorised the reverse flow stemmed from the enlarged diameter rather than the other way round. A relation between reverse systolic flow and increased aortic diameter has concurrently been observed in research investigating BTAI patients treated with TEVAR. A paper by Gil‐Sala et al. (2021), investigating post‐TEVAR changes in aortic diameter, particularly in the ascending aorta (excluding the aortic root), described the influence of TEVAR in systolic flow reversal. Gil‐Sala et al.’s study documented a more than a two‐fold increase in systolic flow reversal in the ascending aorta of TEVAR‐treated BTAI patients, reaching up to five times higher in sections of the aortic arch compared to controls. Even after adjusting for cardiovascular risk factors such as hypertension, dyslipidaemia, and smoking status, TEVAR‐treated patients showed a significantly larger ascending aortic diameter than healthy controls, with an average increase of 3.3 mm (11%) in diameter (*P* = 0.005).

The observed increase in reverse systolic flow in the ascending aorta can be attributed to the previously described pronounced transition from the native aorta proximally and the foreign endograft distally. This idea is particularly supported by the observation of Canaud et al. (2015) that the proximal landing zone, where this transition occurs, was found to enlarge post TEVAR in BTAI patients, especially in those under the age of 30. A theory by Vallerio et al. (2019) further explains the observed remodelling in the ascending aorta. Vallerio et al. posit that the introduction of a rigid endograft into the aorta leads to an abrupt increase in aortic stiffness and subsequent raises in blood pressure as previously described. This elevation in blood pressure triggers vascular remodelling, particularly affecting upstream structures where pressures are highest, contributing to the observed dilatation in the ascending aorta. A similar study by Hiraoka et al. (2020) analysed non‐grafted sections of the ascending aorta following TEVAR through 4D flow magnetic resonance imaging in three patients and speculated that the observed increase in diameter may be attributed to the wall shear stress (WSS) (force per unit area applied by a moving fluid along the surface of the vessel) resulting from the flow alterations induced by the graft. Hiaroka et al. described how the WSS itself as well as WSS‐induced remodelling could account for the observed increases in aortic diameter.

The study of Gil‐Sala et al. (2021) also assessed aortic length and found that only the ascending aorta was significantly affected, with an average increase of more than 14 mm (17%) in length (*P* < 0.001) compared to healthy controls. This elongation, combined with the increase in diameter, resulted in a notably larger aortic volume in the TEVAR group. These results were corroborated by Bero et al. (2020), who observed an average increase of 5.7 mm in length and 1.5 mm in diameter in the ascending aorta when comparing pre‐ and post‐TEVAR CTAs. Bero et al. also found that both the proximal and the distal seal zone diameters, as well as the ascending aorta length, continued to expand significantly from the initial TEVAR to follow‐up scans 1.5 years later. Changes in aortic size have been linked to an increased risk of aortic rupture, dissection and mortality, collectively known as aortic adverse events (AAE). In a study by Wu et al. (2019), the researchers investigated the association between ascending aorta length (AAL) and the risk of AAE. They found that patients with an AAL of 13 mm or more experienced a mean yearly rate of AAE that was five times higher than those with an AAL of less than 9 mm. This confirmed that aortic increases in size as seen following TEVAR pose great risk to affected individuals. The heightened risk of AAE has also been linked to aortic stiffening. Studies like that of Sugawara et al. (2008) suggest that aortic lengthening, driven by aortic wall thinning, leads to increased stiffness, which in turn raises PWV. Other studies describe aortic lengthening as an asymmetrical change, which further disrupts haemodynamics (Kruger et al., 2018). The overall increase in aortic length, therefore, is clinically significant due to the haemodynamic alterations and their associated risks that result as well as the AAE, which can possibly become fatal.

These findings suggest that TEVAR not only immediately alters aortic shape and size but also induces progressive aortic remodelling over time. Callaghan et al. (2018) described the criticality of aortic shape in younger patients for preserving structured blood flow, noting that even gradual, age‐related morphological changes can alter flow dynamics. Consequently, the more pronounced structural changes induced by TEVAR are likely to have a substantially greater impact, significantly disrupting flow patterns within the aorta. Specifically, younger aortas appear to exhibit greater morphological alterations following TEVAR in BTAI patients as evidenced by Canaud et al. (2015). This highlights the substantial effects of TEVAR on aortic morphology and suggests a disproportionate impact on younger patients. Additionally, Callaghan et al. (2018) were the first to describe the predominant influence of stroke volume‐to‐aortic volume ratio in establishing optimal flow conditions. Thus, the previously described aortic volume changes seen in TEVAR patients may directly disrupt the optimal flow present in young healthy aortas.

In addition, Gennai et al. (2020) established a significant correlation between graft oversizing and aortic remodelling, noting that each 10% increase in graft oversizing contributed to a 3.4% increase in the diameter of the proximal landing zone. Oversizing in patients with BTAI, however, can be particularly crucial given that studies have shown aortic size can decrease by more than 15% due to hypovolaemia, leading to potential underestimations of aortic dimensions. Therefore, a strategy that incorporates graft oversizing by 130% is necessary to balance the need for adequate sealing while minimising the risk of excessive aortic dilatation (Bae & Jeon, 2022). This underscores the importance of precise graft sizing to mitigate excessive dilatation and yet reduce the risk of endoleaks, particularly in younger patients who may experience more pronounced morphological changes. Notably, while oversizing has consistently been linked with size alterations, the type of device used did not show a significant influence on aortic size in any of the studies reviewed.

Aortic arch remodelling has also been observed following TEVAR, with the introduction of stiff guidewires and stents during the procedure disrupting the complex geometry of the aortic arch, which features multiple angulated curvatures across three planes. Mestres et al. (2016) examined the remodelling that occurs in the aortic arch following TEVAR, comparing its effects in patients with blunt thoracic aortic injury (BTAI) and those with thoracic aortic aneurysms. Their study cohort included 16 BTAI patients with a mean age of 42.5 years and 20 aneurysm patients with a mean age of 69.5. They observed curvature to only be significantly affected by TEVAR in BTAI in the distal ascending aorta, highlighting a change of −7.5 degrees in angulation in a leftwards and anterior direction. This alteration resulted in an overall less curved distal ascending aorta when analysed in a two‐dimensional *XY* axial plane. The aortic arch was also observed to be less curved (−3.5 degrees) when examined three‐dimensionally, with the BTAI cohort demonstrating a greater decrease in aortic arch angle than the thoracic aortic aneurysm population. Both Gil‐Sala et al. (2021) and Ullery et al. (2018) also observed a decreased angulation in the distal ascending aorta and an increased curvature in the area proximal to the proximal landing zone, as well as an overall reduced curvature in the areas covered by the endograft. The studies describe a general tendency in TEVAR‐induced aortic remodelling, where the aorta transitions from a curved arch to a more squared shape. This occurs due to the device's poor conformation to the natural curvature, with an endograft's tendency to spring back to its original straight shape, exerting pressure on the aortic wall and resulting in straighter lines and sharper angles (Spinella et al., 2018). Reports on the haemodynamic alterations caused by these post‐tevar aortic arch changes are scarce. We theorise that the sharper angles observed in the aortic‐endograft transition areas can further contribute to the localised obstruction previously described in these areas, contributing to extended haemodynamic changes which have previously been tied to the endograft and aortic conformity and size mismatch.

Research investigating geometrical alterations to the aorta in BTAI patients following TEVAR has revealed significant changes, particularly in the ascending section, where both length and diameter progressively increase over time. These changes are influenced by factors like oversizing, with the proximal and distal seal zones showing continued physiological changes. Notably, these geometric transformations impact haemodynamics in the region, underscoring the importance of continuous monitoring to assess their implications for patient outcomes.

### 2.3 Cardiac remodelling

The haemodynamic changes following TEVAR, including increased PWV, increased systolic reverse flow, increased vascular stiffness and reduced maximum velocity, have significant implications for cardiac function. These changes perturb the delicate balance maintained by baroreflex sensitivity, ventriculo‐arterial coupling, and autoregulation of organ perfusion. One prominent example is the rise in left ventricular afterload, driven by elevated total vascular resistance. This increase in vascular resistance stems from loss of compliant buffering in the proximal aorta which reduces maximum velocity and heightens systolic reverse flow, both of which contribute to a higher workload for the heart. This heightened afterload demands greater force during left ventricular contraction to propel blood through the stiffer aorta, over time, contributing to ventricular hypertrophy, an adaptive, but ultimately maladaptive, thickening of the Left ventricular wall. This was seen in the study of Kamenskiy et al. (2020) where BTAI patients treated with TEVAR showed an increase in left ventricular mass and left ventricular mass index by a mean of 35 g and 13.13 g/m^2^ at a rate of 10.03 ± 12.79 g/year and 6.25 ± 10.28 g/m^2^/year, respectively, while control patients’ left ventricular features remained the same. A similar study by Youssef et al. (2020) observed no significant changes in left ventricular mass; however, they noted that 60% of patients who developed postoperative hypertension exhibited Grade I diastolic dysfunction.

Additionally, the increased PWV disrupts the timing of reflected pressure waves, raising systolic blood pressure and impairing coronary perfusion (Tzilalis et al., 2012). This haemodynamic shift exacerbates the risk of coronary ischaemia, especially in patients with existing coronary artery disease, as myocardial oxygen supply diminishes (Van Popele et al., 2001). Currently, no studies have directly examined the effect of TEVAR on coronary perfusion in BTAI patients. Despite BTAI patients often being younger and potentially better able to adapt physiologically, there remains a notable gap in the literature concerning this specific impact. Further research is needed to understand how TEVAR may influence coronary perfusion in this population.

### 2.4 Remodelling‐associated TEVAR complications

The physiological remodelling post‐TEVAR in BTAI patients is related to the complications seen in these procedures.

The literature has a general consensus for the lengthening and broadening of the aorta post‐TEVAR. Changes in aortic length can contribute to endograft migration. In a study by Geisbusch et al. (2019), which investigated endograft migration following TEVAR, aortic lengthening was identified as a significant predisposing factor for graft migration, with a *P*‐value of 0.03. Endograft migration in itself is associated with endoleaks, a serious complication that can result in the need for reintervention and in rare cases cause death (Skrypnik et al., 2024).

The aortic and endograft compliance mismatch previously described has also been correlated to aortic wall weakening contributing to major complications such as retrograde aortic dissection, aneurysmal dilatation, rupture and stent‐graft‐induced new entry tears (Nauta et al., 2016).

Aortic lengthening and compliance mismatch can also destabilise baroreceptor function, affecting short‐term blood pressure regulation and promoting sympathetic activation. These disruptions contribute to the onset of postoperative hypertension, which in turn exacerbates afterload and perpetuates maladaptive remodelling across the cardiovascular system.

TEVAR devices for this relatively young cohort should adapt to their physiological requirements so that endografts are more compliant, causing less structural and haemodynamic changes, to prevent the arising of cardiovascular issues earlier on. Sultan et al. (2022) poses that a solution in the shorter term would be to reduce stent length in the aorta, as they observed better cardiovascular outcomes after using their staged hybrid single lumen reconstruction (TIGER) protocol before TEVAR, which used a shorter stent. They also described the long term solution to be the use of improved stent‐graft materials and designs, as materials such as expanded polytetrafluoroethylene and polyester are stiff and do not replicate the aortic properties effectively. The *ex vivo* porcine study of Nauta et al. (2016) also described how shorter endografts could benefit BTAI patients, after observing that the PWV increase correlated on aortic length coverage. Adams and Kerns (2014) also posed the need of shorter devices so as to reduce aortic coverage, and described the potential necessity to be able to reconstrain and redeploy endografts so as to adapt to physiological changes. It is clear that in this rapidly advancing field, there is an urgent need for the development of endografts specifically designed to meet the physiological requirements of younger patients; this shift from structural repair to physiological preservation may be critical in ensuring TEVAR evolves from a life‐saving intervention to a genuinely restorative one.

## 3 CLINICAL OUTCOMES OF TEVAR IN BTAI: AN OVERVIEW

As aforementioned, given the considerable utilisation of TEVAR over the past decade as the preferred treatment option for BTAI, it is important to thoroughly appraise both its short‐ and long‐term outcomes, particularly considering the younger patient population usually afflicted. Findings in the published literature reveal significant cardiac and aortic modifications, displaying clinical heterogeneity (Evans et al., 2022). Analysing TEVAR outcomes, with a particular focus on mortality, re‐intervention, endoleak and cerebrovascular accidents, is consequently vital for improving the standard of care. This not only guides healthcare practitioners in optimising treatment strategies but also contributes to the broader understanding of TEVAR's role in addressing the long‐term consequences of BTAI.

### 3.1 Mortality

TEVAR for BTAI has demonstrated highly favourable mortality and survival results, showing its effectiveness and superiority in comparison to OSR. For example, Cheng et al. (2019) revealed lower in‐hospital mortality risk in the TEVAR cohort (9.0%) compared to the OSR cohort (27.0%) after propensity score matching. Recent studies, including Farber et al. (2022), reinforce these positive trends, demonstrating significant improvements in mortality outcomes over both short‐ and long‐term intervals. Farber reported freedom from all‐cause mortality at 95% and 89% at 1 month and 5 years, respectively. Mufty et al. (2019) similarly showed 100% survival at 6 months, 1 year and 2 years, with no device‐related complications. Buczkowski et al.’s (2023) recent findings indicated excellent early mortality of 1.3%, and sustained long‐term survival probabilities of 86.4%, 80.0% and 76.6% at 1, 5 and 10 years, respectively. Furthermore, the TRANSFIX study, with 50 BTAI patients, reported a 2% 30‐day mortality rate and high freedom from all‐cause (89.3%) and BTAI‐related (97.6%) mortality at 2 years (Jubouri et al., 2024). Gennai et al.’s (2023) meta‐analysis also demonstrated a late survival rate of 95.6%. Two 5‐year studies reported survival rates of 85.2% and 89%, with no repeat interventions required. Finally, Agostinelli et al.’s (2019) 20‐year experience showed excellent estimated survival rates at 5 and 10 years (92% and 87%).

On the other hand, Conrad et al. (2010) showed survival rates of 86%, 75%, 70% and 65% at 6 months, 1 year, 2 years and 5 years, respectively. Martin et al. (2017) also reported lower 5‐ and 10‐year survival rates of 81.6% and 75.2%, potentially attributed to follow‐up challenges and non‐compliance in a younger trauma population. Azizzadeh et al.’s (2014) initial experience in 2009 showed no mortality among 27 patients, but their 2013 update reported a 5% in‐hospital mortality in a larger cohort of 82 patients. Prendes et al.’s (2021) two‐decade experience revealed a 17.5% in‐hospital mortality rate for predominantly Grade 3 or 4 BTAI patients. The extensive American College of Surgeons‐verified Level I trauma centre study, spanning 6 years and 382 TEVAR‐treated BTAI patients, showcased in‐hospital and aorta‐related mortality rates of 18.8% and 6.5%, respectively (Jubouri et al., 2024). These high rates could be correlated with the grade of injury, being respectively 12.8% and 0 for Grade 1, 20.6% and 2.9% for Grade 2, 16.7% and 5.2% for Grade 3, and 50% and 46.4% for Grade 4 BTAI. Multivariate logistic regression analysis identified BTAI grade as an independent predictor of mortality, underscoring the nuanced relationship between injury severity and survival outcomes in TEVAR‐treated BTAI patients (Jubouri et al., 2024).

### 3.2 Cerebrovascular accidents

Cerebrovascular accidents (CVAs), commonly reported as strokes, represent significant complications in patients undergoing TEVAR for BTAI. Perioperative mortality rates associated with CVA range from 7% to 9%, underscoring the gravity of this concern, with strokes being a primary concern (Jubouri et al., 2024). Nevertheless, studies have reported significantly low incidence of CVA after TEVAR for BTAI. Additionally, the incidence of postoperative stroke and paraplegia from spinal cord ischaemia has been reported at as low as 1% and 1.4%, respectively (Harky et al., 2020). However, given the potential impact on outcomes, meticulous assessment and effective management of CVA risk factors are imperative in this clinical context.

In a cohort of 60 BTAI patients, Martin et al. (2017) reported a remarkable success rate with no instances of CVA or paraplegia during a mean follow‐up of 5 years, reaching a maximum of 14 years. Likewise, Spiliotopoulos et al. (2014), demonstrated excellent outcomes in their cohort of 76 patients with no cerebrovascular accidents, paraplegia or paraparesis. Similarly, the aforementioned Farber et al. (2022) also observed minimal stroke incidence in a cohort with partial or complete left subclavian artery coverage. Diaz et al. (2021) reported a 1.4% occurrence of postoperative paraplegia from spinal cord ischaemia after TEVAR for BTAI, while Buczkowski et al. (2023) documented transient paraparesis in 1.3% of cases early after the procedure. Abdou et al. (2021) reported a postoperative stroke incidence of 2.8%, emphasising the need for vigilance against cerebrovascular events in TEVAR for BTAI. The study advocates for collaborative efforts between surgeons and critical care providers to enhance postoperative neurological outcomes. Consequently, a thorough preoperative neurological evaluation and vigilant postoperative monitoring are crucial components for identifying and managing CVA risks effectively.

Finally, a recent study published in the *European Journal of Trauma and Emergency Surgery* delved into the long‐term outcomes and health‐related quality of life of BTAI patients undergoing TEVAR (Hundersmarck et al., 2020; Mufty et al., 2019). The research underscored the necessity for further exploration into TEVAR's patency and complications in the BTAI context, particularly concerning severe cerebral ischaemia following secondary rupture. Strategies such as the incorporation of embolic protection devices during the procedure are proposed to mitigate the risk of stroke. The diverse findings from various studies underscore the complexity of the risks associated with CVA, emphasising the need for ongoing research and strategic approaches to enhance patient outcomes and minimise neurological adverse events.

### 3.3 Re‐intervention

Extensive variability exists in reported reintervention rates for BTAI patients undergoing TEVAR, ranging from 2.1% to 19.6% (Gennai et al., 2023). This trend is evident in the literature whereby the increase in the number of patients undergoing endovascular interventions for BTAI has been dramatic, from 6.5% to 86.5% over 6 years, according to Ultee et al. (2016). The more recent meta‐analysis by Gennai et al. (2023) featured a mean follow‐up period of 8.2 years for 389 BTAI patients who survived more than 30 days post‐TEVAR (Gennai et al., 2020; Gennai et al., 2023). Their results showed a 2.1% late reintervention, with only 0.1% occurring after 5 years. Similarly, the TRANSFIX prospective, multicentre, non‐randomized, single‐arm study of 50 BTAI patients with a mean follow‐up of 21 months reported a freedom from reintervention was significantly low with 85.9% at 1 year and 83.3% at 2 years ((Jubouri et al., 2024; Starnes et al., 2020). Evans et al. (2022) revealed findings from their 11‐year Canadian retrospective cohort study encompassing 427 BTAI patients, with 105 individuals (24.6%) opting for TEVAR. Following a median follow‐up duration of 3 years, 5.7% (*n* = 6) of these patients required reintervention. Two 5‐year studies reported survival rates of 85.2% and 89%, with no further interventions required (Jubouri et al., 2024). However, Stueur et al. (2015) reported an interesting finding, with 16% (*n* = 12) of their 74 patients needing reintervention within the first postoperative year.

Additionally, long‐term studies such as Mufty et al. (2019) and Bero et al. (2020), have investigated the patterns of early and late re‐interventions. Interestingly, Mufty's study reported no early re‐interventions, given their reported incidences of endoleak (6.7%), infolding (3.7–6.7%), left arm ischaemia (2.7%) and fibrous dysplasia (1.3%). On the other hand, Bero et al. (2020) found a correlation between the need for secondary intervention and endograft mural thrombus (*P* < 0.05). Another long‐term study demonstrated a high freedom from reintervention rate of 97.4% up to 5 years after TEVAR. In the study by Azizzadeh et al. (2014), out of their 82 patients, only four (4.9%) underwent reintervention (open). Agostinelli et al. (2019), with a 20‐year experience and 35 patients, reported excellent estimated freedom from reintervention rates of 96% at 5 years and 91% at 10 years, with no aorta‐related deaths. Martin et al. (2017) described no reinterventions were required in their cohort of 60 patients over 14 years. This suggests that the need for reintervention following TEVAR in BTAI patients is rare, thus supporting the effectiveness of TEVAR.

In conclusion, the literature consistently supports the infrequent need for reintervention following TEVAR in BTAI patients, highlighting its effectiveness as a long‐lasting therapeutic approach. When compared to other aortic conditions, TEVAR for BTAI has shown significantly lower rates of reintervention, making it a promising treatment option. This is in contrast to findings in aortic dissections or aneurysms, where reintervention rates tend to be higher, possibly due to the presence of persistent vascular disease within the aortic wall (Ho et al., 2021). As the field continues to evolve, further research and collaboration are needed to refine our understanding.

### 3.4 Endoleak

The literature frequently reports endoleak, bird beaking (improper proximal positioning of the endograft), endograft collapse, thrombosis and fracture as the most common adverse events related to grafts in BTAI cases (Gennai et al., 2023). However, it is important to note that these complications occur rarely in the general context of endovascular repair (Xu et al., 2017). The study of Piffareretti et al. (2013) involving 46 consecutive cases, neither endoleaks nor modifications of the native aorta were detected. Contrarily, Fontana et al. (2018) reported in their retrospective study that 8.7% (*n* = 2) of cases exhibited endograft‐related complications, with one instance of endoleak identified at 2 and 4 months post‐TEVAR. In a larger cohort of 453 patients, however, Dubose et al. (2015) observed a 2.5% incidence of endoleaks. Similarly, in a retrospective study of 99 patients, Kim et al. (2019) identified only one case of type Ia endoleak occurring at 18 months postoperatively.

While these findings contribute valuable insights, the existing literature underscores the need for more comprehensive research focusing on aortic remodelling and endoleak outcomes. These factors are crucial, given their potential association with an increased incidence of reinterventions.

## 4 CONCLUSION

Overall, TEVAR remains the preferred treatment modality for BTAI, offering better survival and fewer complications in the short term. Yet, careful long‐term follow‐up is essential, particularly for younger patients, to monitor for potential adverse outcomes related to the endograft and its interaction with the cardiovascular system. Future research should focus on improving the adaptability and longevity of stent grafts for younger populations, aiming to mitigate long‐term complications while preserving the initial benefits of TEVAR.

## AUTHOR CONTRIBUTIONS

Conception and design: Matti Jubouri, Mohamad Bashir. Analysis and interpretation or Data collection: All authors. Writing the manuscript: Marco David Bokobza De la Rosa, Matti Jubouri, Thurkga Moothathamby, Mohamed Refaie. Critical revision: Ali Murtada, Idhrees Mohammed, Ian M. Williams, Damian M. Bailey, Mohamad Bashir. All authors have read and approved the final version of this manuscript and agree to be accountable for all aspects of the work in ensuring that questions related to the accuracy or integrity of any part of the work are appropriately investigated and resolved. All persons designated as authors qualify for authorship, and all those who qualify for authorship are listed.

## CONFLICT OF INTEREST

D.M.B. is Editor‐in‐Chief of *Experimental Physiology*, Chair of the Life Sciences Working Group, member of the Human Spaceflight and Exploration Science Advisory Committee to the European Space Agency and member of the Space Exploration Advisory Committee to the UK and Swedish National Space Agencies. D.M.B. is also affiliated to Bexorg, Inc. (USA) focused on the technological development of novel biomarkers of cerebral bioenergetic function and structural damage in humans.

## Data Availability

The evidence used to support this review are publicly available in electronic databases such as PubMed, Google Scholar, Ovid, Scopus and Embase.
